# Neuromelanin is an immune stimulator for dendritic cells *in vitro*

**DOI:** 10.1186/1471-2202-12-116

**Published:** 2011-11-15

**Authors:** Uwe Oberländer, Katrien Pletinckx, Anja Döhler, Nora Müller, Manfred B Lutz, Thomas Arzberger, Peter Riederer, Manfred Gerlach, Eleni Koutsilieri, Carsten Scheller

**Affiliations:** 1 University of Würzburg, Institute of Virology and Immunobiology, Würzburg, Germany; 2 University of Munich, Institute of Neuropathology, Munich, Germany; 3 University of Würzburg, Clinical Neurochemistry (National Parkinson Foundation Center of Excellence Research Laboratory), Clinic and Policlinic of Psychiatry, Psychosomatics and Psychotherapy, Würzburg, Germany; 4 University of Würzburg, Laboratory of Clinical Neurobiology, Department of Child and Adolescent Psychiatry, Psychosomatics and Psychotherapy, Würzburg, Germany

## Abstract

**Background:**

Parkinson's disease (PD) is characterized at the cellular level by a destruction of neuromelanin (NM)-containing dopaminergic cells and a profound reduction in striatal dopamine. It has been shown recently that anti-melanin antibodies are increased in sera of Parkinson patients, suggesting that NM may act as an autoantigen. In this study we tested whether NM is being recognized by dendritic cells (DCs), the major cell type for inducing T- and B-cell responses *in vivo*. This recognition of NM by DCs is a prerequisite to trigger an adaptive autoimmune response directed against NM-associated structures.

**Results:**

Murine DCs were treated with NM of substantia nigra (SN) from human subjects or with synthetic dopamine melanin (DAM). DCs effectively phagocytized NM and subsequently developed a mature phenotype (CD86^high^/MHCII^high^). NM-activated DCs secreted the proinflammatory cytokines IL-6 and TNF-α. In addition, they potently triggered T cell proliferation in a mixed lymphocyte reaction, showing that DC activation was functional to induce a primary T cell response. In contrast, DAM, which lacks the protein and lipid components of NM but mimics the dopamine-melanin backbone of NM, had only very little effect on DC phenotype and function.

**Conclusions:**

NM is recognized by DCs *in vitro *and triggers their maturation. If operative *in vivo*, this would allow the DC-mediated transport and presentation of SN antigens to the adaptive immune system, leading to autoimmmunity in susceptible individuals. Our data provide a rationale for an autoimmune-based pathomechanism of PD with NM as the initial trigger.

## Background

Parkinson's disease (PD) is a progressive neurodegenerative disorder characterized at the cellular level by a destruction especially of neuromelanin (NM)-containing dopaminergic cells and a profound reduction in striatal dopamine. NM accumulates in the cytoplasm of dopaminergic neurons starting within the first 3-5 years after birth [1-3]. NM concentration increases with age and its optical density has been shown to increase until 60 years of life [4]. In patients with juvenile PD as well as with idiopathic and MPTP-induced PD, NM also accumulates in the extracellular space of substantia nigra (SN) [5-7].

Exracellular NM does not behave passively. Both protective and toxic effects have been reported (reviewed by Zecca [8,9]). The protective role is most notably through its ability to trap free radicals and toxins [8]. Toxic effects of NM are mainly due to NM highly-complexed with iron [10,11]. *In vitro *NM activates microglia by triggering NK-κB activation and the release of the proinflammatory cytokines TNF-α and IL-6 [12]. When injected into the brains of rats, human NM triggers neuroinflammation and neurodegeneration [13,14], suggesting a proinflammatory role for NM.

The primary etiological factor for PD is still unknown. Several hypotheses have been proposed [15-18]. Autoimmune processes in PD have been suggested previously [19,20]. Autoantibodies directed at neuronal structures have been found in sera of PD patients [21-25]. In an interesting study brains of PD patients exhibited an IgG binding on dopamine neurons, which was positively correlated with the number of HLA positive microglia [26]. Moreover, in the same study the low affinity activating IgG receptor FcγRIII was expressed on cells morphologically resembling lymphocytes. In a recent publication by Double et al., sera from subjects with clinical PD were found to display significantly enhanced IgG-levels specific for melanin derived from catecholamines, a structural component of NM [16]. Moreover, complement binding to NM in brains from PD patients supports the idea of immunologic clearance of NM in PD [7]. These data suggest the possibility of a specific autoimmune response against NM in PD patients.

Dendritic cells (DC) are professional antigen-presenting cells able to initiate primary T-cell mediated immune responses [27]. In a so-called immature state their main function is the uptake and processing of antigens. Once they become activated (either by proinflammatory cytokines or pathogens), DCs migrate into the draining lymph nodes (LN) to present the antigen to naïve T- and B-cells [27]. If T- or B-cells specific for this antigen enter the LN, the cells become primed to exert their effector functions once they re-encounter the antigen in the peripheral tissue. In contrast, microglia are tissue-resident cells that are not able to migrate into lymphoid tissues to start an adaptive immune response. They are specialized to present antigens to already activated, infiltrating T-cells. Hence, a de novo initiation of an adaptive immune response against an immunogen within the brain requires DC activation, whereas microglia activation is in this context a downstream event in order to direct the T- and B- cell response to the site where the antigen is located. DCs are spare in the healthy CNS. However, they do accumulate in the CNS parenchyma in neuroinflammation and CNS autoimmune disease following monocyte invasion, from which they differentiate [28-33].

In this study we hypothesize that extracellular NM may stimulate DC maturation and thereby promote NM-associated antigen presentation in an autoimmunogenic context. This would reflect the initiating step of an autoagressive activity against antigens from SN. Therefore, we investigated whether NM can be recognized and taken up by DCs and subsequently explored the capability of NM to mature/activate these cells phenotypically and functionally for T cell activation. Using synthetic dopamine melanin (DAM), we investigated the role of the domanine melanin "backbone" found in NM in DC activation.

## Results

### Dendritic cells phagocytose NM and DAM

**Figure 1:**
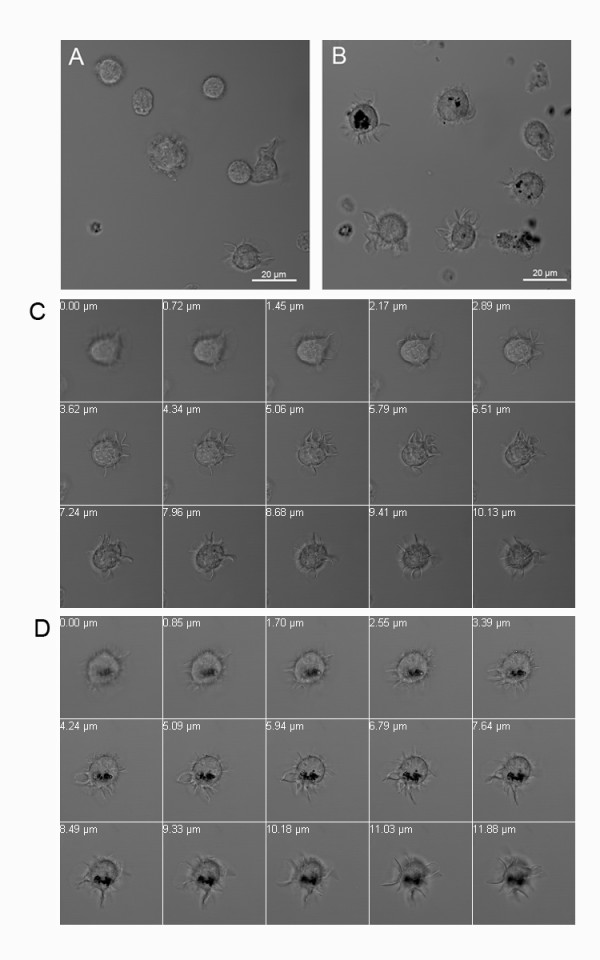
**Dendritic cells (DC) phagocytose neuromelanin (NM)**. Cells were cultured for 48 h in the absence (A, C) or presence (B, D) of neuromelanin (NM) and analyzed by differential interference contrast (DIC) microscopy. A, B: Overview of cultures. C, D: z-stack analysis of a single DC from bottom to top. Z-stack distances were 0.70-0.80 μm.

**Figure 2:**
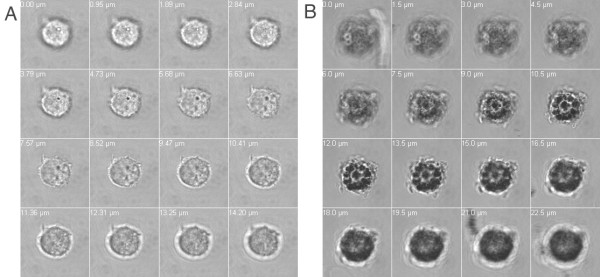
**Dendritic cells (DC) phagocytose synthetic dopamine melanin (DAM)**. Cells were cultured for 48 h in the absence (A) or presence (B) of DAM. Differential interference contrast (DIC) microscopy with z-stack analysis of DCs from bottom to top. Z-stack distances were 1.5 μm.

In order to assess whether DCs recognize NM as a potential antigen we coincubated DCs with NM and studied phagocytosis by differential interference contrast microscopy. NM was visible as small black granules of 0.5-5 μm size (Figure 1). After 24 hours of co-incubation with DCs NM colocalized with DCs in the culture plate (Figure 1). A z-stack analysis revealed that NM granules were not just attached to the cell surface but were indeed internalized by DCs (Figure 1D). Similarly, DCs also phagocytosed DAM (Figure 2).

### DCs mature in response to NM

**Figure 3:**
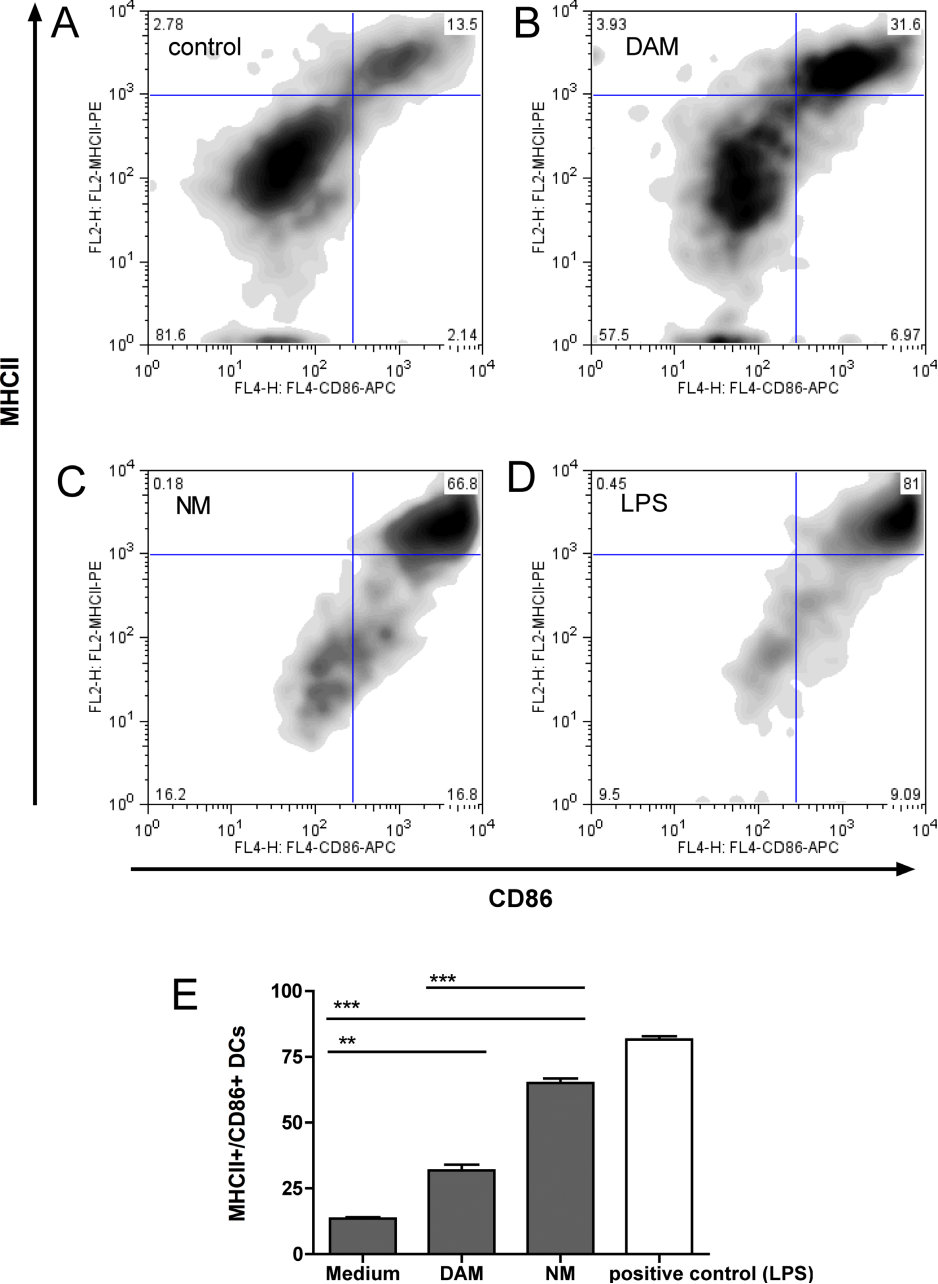
**Neuromelanin triggers dendritic cell (DC) maturation**. Flow cytometric analysis of DC maturation by staining with anti-CD86/anti-MHCII antibodies. A: medium-treated DCs (untreated). B: DAM-treated DCs. C: NM-treated DCs. D: positive control (LPS-treated DCs). A-D: representative experiments from triplicates. E: Illustration of the amount of mature DCs from triplicates (data as mean ± S.E.M.; statistical analysis with one-way ANOVA using Bonferroni multiple comparison as post test; n.s. = non specific (p > 0.05), * p < 0.05, ** p < 0.01, *** p < 0.001).

Phagocytosis of NM resulted within 48 h in maturation of DCs measured by the upregulation of the cell surface molecules MHCII and CD86. Whereas only 13% of the DC population displayed a mature phenotype in the absence of additives in the culture medium, the amount of mature DCs increased to 66.8% following contact with NM (Figure 3A, C and 3E; Bonferroni post hoc test, p < 0.001). DAM triggered comparably lower DC activation (31.6%) compared to NM (Figure 3B and 3E; p < 0.001) but still significantly elevated compared to medium alone (p < 0.01). As a positive control, DCs were treated with LPS (Figure 3D and 3E), a strong activator of DCs. LPS-activation in the experiments displayed in this manuscript should only be regarded qualitatively (instead of quantitatively), as the amount of LPS used in these experiments is in no relation whatsoever to the amount of NM or DAM, except for the fact that it was titrated in order to provoke a response. We therefore did not apply statistical analysis comparing the magnitude of the LPS response with other treatments.

### NM generates a functional activation of DCs

**Figure 4:**
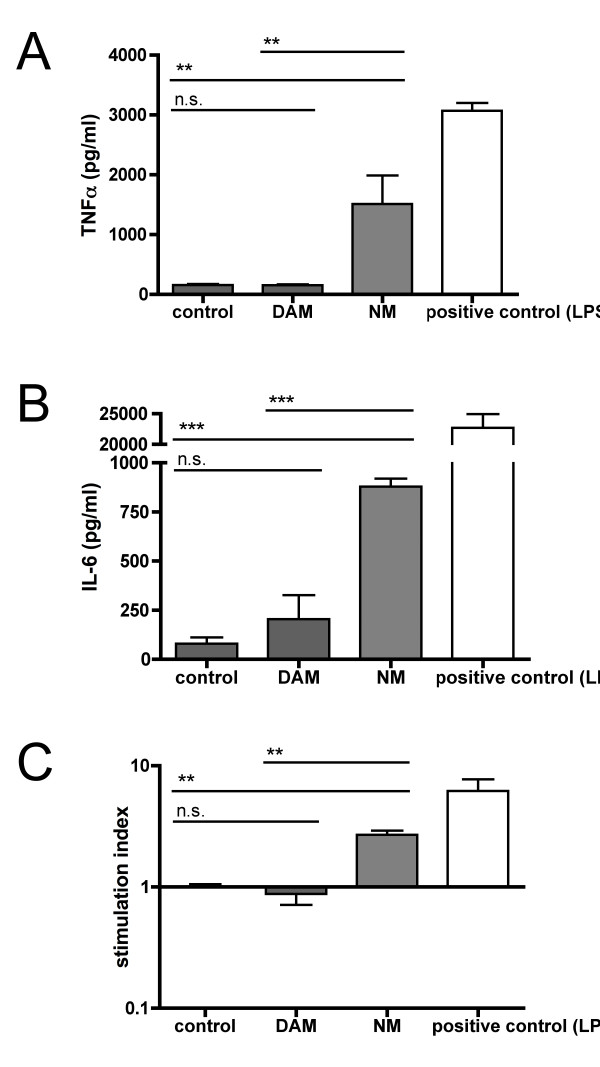
**NM-mediated dendritic cell (DC) activation is functional and independent of the melanin backbone**. A, B: DCs were cultured for 48 h in the presence of medium alone, DAM, NM, or LPS (positive control). The amount of proinflammatory cytokines in the cell culture supernatants was determined by ELISA. A: TNF-α. B: IL-6. C: DCs treated for 48 h with medium alone, DAM, NM or LPS (positive control) were cocultured with allogenic T cells. A-C: Data from 2 independent experiments with triplicates and quadruplicates as mean ± S.E.M.; statistical analysis with one-way ANOVA using Bonferroni multiple comparison as post test; n.s. = non specific (p > 0.05), * p < 0.05, ** p < 0.01, *** p < 0.001).

Following 48 h exposure to NM, DCs released significantly higher amounts of proinflammatory cytokines such as TNF-α and IL-6 compared to cells treated with medium alone (p < 0.01 and < 0.001, respectively), demonstrating that NM-triggered DC maturation generated functional cells (Figure 4A, B). No additional cytokine release compared to cells treated with medium-alone was detected in cells treated with DAM.

In order to test whether NM-stimulated DCs can activate T cell proliferation (a necessary event in the translation of a DC signal into a T cell response), we performed a mixed lymphocyte reaction (MLR) in which T cells isolated from allogenic donor mice were cocultured with NM- or DAM-treated DCs. As depicted in Figure 4C, NM-treated DCs were able to trigger a proliferative T cell response (p < 0.001 compared to untreated cells). In contrast to the effects triggered by NM, no T cell proliferation could be detected following incubation with DAM.

The experiments displayed in Figure 4 not only demonstrate that NM-triggered DC activation generates completely functional DCs (cells that are able to migrate to the lymph nodes and present antigens to T cells and that also have the capacity to trigger proliferative T cell responses), but also narrows the number of NM-compounds that could actually be the trigger of it: As synthetic DAM, which lacks many of the compounds found in NM (lipids, proteins) failed to triggered any cytokine or proliferative response at all, the dopamine melanine itself is probably not the stimulus for these events. However, the dopamine melanine "backbone" of NM could play an important role in efficient uptake of NM by DCs, as we observed that DAM alone is being recognized and phagocytosed very efficiently by DCs (Figure 2).

### NM preparations are free of endotoxin contamination

**Figure 5:**
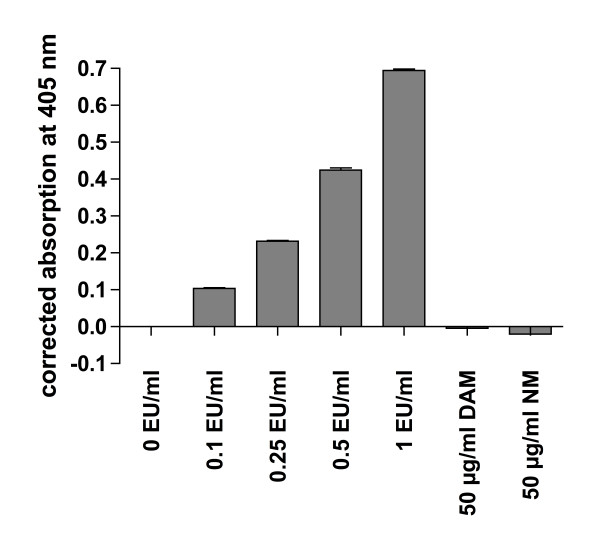
**Negative test of LPS contamination**. The DAM- and NM-preparations used in this study were tested with an endpoint chromogenic LAL-assay for LPS contamination at a concentration of 50μg/ml and analyzed at a wavelength of 405 nm. LPS standards with a concentration range between 0.1-1.0 EU/ml were tested positive, whereas the DAM and NM-preparations were tested negative for LPS.

DCs are very sensitive towards LPS (which for this reason was used as a positive control in the experiments depicted above). In order to exclude a potential endotoxin contamination of the DAM and NM preparations used in our experiments, we tested the DAM- and NM-suspensions for LPS using a commercially available LAL assay. No traces of LPS were detected in the DAM- and NM-suspensions used for our experiments (Figure 5). These results suggest that the DC-stimulating effects are intrinsic for NM and were not caused by a potential contamination with endotoxin.

## Discussion

Our *in vitro *study shows that a) extracellular NM is being phagocytosed by DCs and b) NM triggers maturation and functional activation of these cells. If operative *in vivo*, this would inevitably cause a transport of extracellular NM from the brain into cervical lymph nodes, resulting in a presentation of NM to naïve T- and B-cells in a highly immunogenic context. This may eventually lead to the development of an autoimmune disease directed at NM-associated antigens. Our study therefore offers a first evidence for a NM-driven autoimmune-based pathogenesis of PD with NM as the initial trigger of a DC-mediated autoantigen presentation.

Although an autoimmune-based pathogenesis of PD that targets NM has yet to be conclusively demonstrated *in vivo*, a number of previous findings can now be put into a broader perspective: Sera from subjects with clinical PD display enhanced IgG-levels to catecholeamine-based melanins, the structural basis of the NM present in the pigmented neurons of the human SN [16], indicating an immune response directed against NM itself. In line with this, opsonization of NM with C1q - a complement factor involved in the classical complement pathway that recognizes antigen-bound IgG and IgM - has been shown on the surface of extracellular NM in post-mortem brains of PD-patients [7]. Alternatively (or additionally), DC-phagocytosis of NM loaded with resident peptides or proteins (the high affinity of NM to peptides has been shown before [34]) may initiate the presentation of formerly-unrecognized autoantigens to T- and B-cells in a "Trojan Horse way", triggering an adaptive autoimmune response directed at proteins primarily unrelated to NM. In this regard Orr *et al*. found in post-mortem sections from PD patients - but not from age-matched controls - IgG antibodies directed at pigmented dopamine neurons and auto-IgG binding colocalized with immunostaining for Lewy bodies [26].

Mature DCs loaded with autoantigens have been shown to be able to induce autoimmunity against the loaded antigen in mice [35]. Although DCs seem to be rare in the healthy brain, myeloid-derived DCs infiltrate the brain during neuroinflammation [28-33] so that DCs are likely to encounter extracellular NM also *in vivo*.

Our data indicate that the DC-activating properties of NM are conferred by its peptide or lipid components (but not by the dopamine melanine backbone). Although the primary repertoire of endogenous lipids and peptides is unlikely to be able to activate DCs, oxidative modifications of endogenous molecules are being discussed to confer DC-maturating activity by an "altered-self" mechanism [36]. Such DC-maturating activity has already been described for oxidized lipophosphatidylcholine (LPC) found in low-density lipoprotein (LDL), which potently triggers DC maturation and activation [37,38]. In a similar way, oxidized protein or lipid contents of NM could have triggered DC maturation in our assay system. In line with this, post-mortem studies with brains from PD patients revealed increased lipid-peroxidation in SN [10,39].

Our findings that NM activates DCs are in line with previous reports that NM can activate microglia both in vitro [14] and in vivo when injected into the rat brain [13,14]. Microglia and DCs are both myeloid antigen presenting cells that are closely related, albeit they fulfill completely different but also cooperative functions during an immune response. Whereas activated DCs will leave the tissue and transport the antigen to the lymph nodes in order to *initiate *an adaptive immune response, activated microglia reside in the tissue and present the antigen to DC-primed infiltrating T cells in order to *direct *the immune response to the tissue in which the antigen was found. Therefore, combining the results of the former study on microglia with our results opens a new autoimmune scenario: NM not only causes local inflammation (activation of microglia) but may also trigger an adaptive immune response directed at NM itself via activation of DCs. Our findings therefore add a potential third characteristic to NM in relation to PD: whereas NM has previously been discussed to be either neuroprotective or neurotoxic (and the inflammatory activity of NM on microglia would be a neurotoxic characteristic in the broadest sense) our findings suggest that it might also be considered as an initial trigger for an adaptive autoimmune response.

## Conclusions

Our data suggest that extracellular NM may be the initial trigger for an adaptive autoimmune response relevant for PD via activation of DCs. Whatever the antigen(s) recognized in an autoimmune etiology of PD may be, our data now offers an explanation for the initial trigger of the autoimmune response against SN-antigens, i.e. the activation of DCs by a substance exclusively found in the affected areas, the NM.

## Methods

### NM preparation

Human SN tissue was obtained from the "Austrian-German-Brain-Bank" in Würzburg. The use of *post mortem *human brain tissue was approved by the Ethics Committee of the University Clinics of Würzburg. The SN was dissected from *post mortem *brains of subjects with no history of neurological, neurodegenerative or psychiatric diseases within 36 h of death on a cool plate (-15°C). Only cases with a macroscopically regular pigmentation of the SN and a post mortem delay of less than 36 h were selected for preparation. The SN was dissected from transversally cut midbrain slices on a cool plate (-15°C). NM was isolated according the method previously published [40]. The prepared NM was resuspended in PBS at a concentration of 5 mg/ml by pipetting.

### Dopamine melanin (DAM) synthesis

Synthetic dopamine melanin (DAM), a widely used model of human NM (see for example, [41]) was produced by incubation for 2 weeks of 1 mM dopamine hydrochloride with 0.1 mM cupric sulfate pentahydrate in phosphate buffered saline (pH 7.4). The resulting solid oxidation product was then washed in distilled water and centrifuged four times before being lyophilized. DAM was sonicated in phosphate-buffered saline to produce a suspension of fine, homogenous granules at a concentration of 1 mg/ml. All chemicals were obtained from Sigma-Aldrich, Germany.

### Confocal Microscopy

Cells were seeded onto μ-slide VI (ibidi, Germany). Images were obtained using a Zeiss 510 Meta confocal Microscope (Zeiss, Germany) with a 63 × objective (NA1.4). Z-stack analysis was performed with imaging software SP3.2 (Zeiss, Germany).

### Animals

C57BL/6 (Charles River/Wiga, Sulzfeld, Germany) and BALB/c mice (house bred) were kept under SPF (specific-pathogen free conditions) in our facilities. 4-12 weeks old female mice were sacrificed by cervical dislocation in order to isolate bone marrow and lymph nodes, respectively.

### Bone marrow and lymph node isolation

Femurs and tibiae from C57BL/6 mice were removed and purified from the surrounding muscle tissue by rubbing with Kleenex tissues. Thereafter intact bones were left in 70% ethanol for 2-5 min for desinfection and washed with PBS. Then both ends were cut with scissors and the marrow was flushed out with PBS using a syringe with 0.45 mm diameter needle. Cell clusters were dissociated by vigorous pipetting and cells were washed with PBS. 1-1.5 × 10^7 ^leukocytes were obtained per femur or tibia.

Lymph nodes from BALB/c-mice were removed and a single cell suspension was prepared by grinding the lymph nodes between the rough ends of two sterile slides. The lymphocytes were filtered through a 70 μm Falcon Cell Strain (BD Biosciences, Germany).

### Generation of bone-marrow DCs

DCs were generated from bone marrow of mice as described previously with some modifications [42]. Briefly, bone marrow cells were cultured in 100 mm bacteriological petri dishes (Greiner bio-one, Germany) with R10 (RPMI-1640 supplemented with 10% heat-inactivated fetal calf serum, penicillin (100 U/ml), streptomycin (100μg/ml), L-glutamin (2 mM), and 2-mecraptoethanol (50μM, Sigma-Aldrich, Germany)). All media components were obtained from PAA, Germany.

At day 0 bone marrow cells were seeded at 3 × 10^6 ^cells per dish in 10 ml R10 medium containing 10% GM-CSF supernatant from a cell line transfected with the murine GM-CSF gene [43]. At day 3 and 6 another 10 ml R10 medium containing 10% GM-CSF supernatant was added to the plates. At day 8 cells were used for experiments.

### Treatment of DCs with NM, DAM and LPS

If not indicated otherwise, DCs were treated for 48 h with NM, DAM at a final concentration of 50μg/ml or lipopolysaccharide (LPS) at 1μg/ml (Sigma-Aldrich, Germany) or left untreated in a 24 well plate with 10^6 ^cells/well. NM- and DAM suspensions used in DC stimulation experiments were checked for endotoxin contamination and found negative (cutoff was 0.1 EU/ml; endpoint chromogenic Limulus Amebocyte Lysate (LAL) assay, Lonza, Germany).

### Flow cytometry

DCs were characterized by flow cytometry. 1 × 10^5 ^cells were stained with fluorochrome-conjugated antibodies directed at CD86 (coupled to APC) and MHCII (coupled to PE) (both BD Biosciences, Germany) at pre-titrated concentrations at 4°C for 60 minutes. Cells were fixed with 2% formalin and analyzed using a FACSCalibur (BD Biosciences, Germany). Cell debris was excluded from analysis by FSC-SSC gating. Quadrants were set according to staining patterns with isotype-control antibodies (all antibodies from BD Biosciences, Germany). Flow cytometry data was analyzed using FlowJo software (Tree Star Inc., OR, USA).

### ELISA

Tumor necrosis factor (TNF)-α and interleukin (IL)-6 concentrations were determined from supernatans of NM- or LPS-treated or untreated DCs using the respective OptEIA Kits (BD Biosciences, Germany) according to the instructions of the manufacturer.

### Allogeneic mixed lymphocyte reaction (MLR)

DCs from C57BL/6 mice were treated with NM, LPS or left untreated for 48 h. At day 3, DCs were coincubated with lymphocytes isolated from BALB/c lymph nodes in triplicate cultures (96 well) at 3 × 10^5 ^cells/well and a ratio of 1:1. At day 3, cells were pulsed with 1 μCi/well [^3^H]methyl-thymidine (Amersham Biosciences, Switzerland) over night for 16 h. The plates were harvested onto glassfiber filtermats with an Tomtec harvester and filters counted in a 1450 Microplate Counter (Wallac, Turku, Finland).

### Test of endotoxin contamination

The NM-preparation used for the experiments depicted in this manuscript was tested for potential contamination with endotoxin using a chromogenic *Limulus *amebocytes lysate (LAL) assay according to the instructions of the manufacturer (Lonza, Germany). Briefly, 50μl of NM sample (100 μg/ml), LPS standards (0.1-1.0 EU/ml) or endotoxin-free water were mixed with 100 μl of LAL and incubated for 10 min at 37°C. Substrate solution was added and samples were incubated for an additional 6 min at 37°C. Enzyme reaction was stopped with diluted sulfuric acid and chromogen absorption was measured in a plate photometer at a wavelength of 405 nm. To account for NM-intrinsic absorption at 405 nm, a turbidity control with 100 μg/ml NM without LAL (volume compensated with water) was measured and the value was subtracted from NM-sample absorption at 405 nm.

### Statistical analysis

Statistical analysis was performed using GraphPad Prism software for Macintosh (version 4.0 c). Data are expressed as mean ± S.E.M.. For comparison of values between different treatment groups, one-way ANOVA was used together with Bonferroni multiple comparison as post test; p > 0.05 was regarded as non specific, p < 0.05 was attributed with *, p < 0.01 was attributed with **, and p < 0.001 was attributed with ***.

## List of abbreviations

Parkinson's disease (PD), neuromelanin (NM), dendritic cells (DCs), dopamine melanin (DAM), substantia nigra (SN), interleukin-6 (IL-6), tumor necrosis factor (TNF), major histocompatibility complex (MHC), differential interference contrast (DIC)

## Authors' contributions

UO carried out treatment of DCs with NM, DAM and LPS and performed immunological charactertization of the response of DCs to NM including the MLR. KP and AD carried out bone marrow and lymph node isolation and generation of bone-marrow DCs. NM performed confocal microscopy. MBL participated in study design, supervized DC preparation and helped to draft the manuscript. TA, PR and MG participated in coordination and design of the study and helped to draft the manuscript. EK and CS conceived of the study, participated in all steps of the work and drafted the manuscript. All authors read and approved the final manuscript.
